# Long‐term outcomes of peritoneal dialysis catheters inserted by laparoscopic and percutaneous techniques in a single regional dialysis centre

**DOI:** 10.1111/ans.17644

**Published:** 2022-04-03

**Authors:** Wai Gin Lee, Phuong Uyen Tran, Richard Grills

**Affiliations:** ^1^ Department of Urological Surgery Barwon Health, University Hospital Geelong Geelong Victoria Australia; ^2^ Department of Surgery School of Medicine, Deakin University Geelong Victoria Australia

**Keywords:** laparoscopic insertion, percutaneous insertion, peritoneal dialysis, tenckhoff catheter

## Abstract

**Background:**

Minimally invasive insertion of catheters for peritoneal dialysis can be performed laparoscopically or percutaneously under image guidance. In Geelong (Victoria, Australia) both methods are used. Our aim was to analyse the outcomes of all catheters inserted by both laparoscopic and percutaneous techniques and compare them against published studies from tertiary referral centres.

**Methods:**

Data were collected retrospectively on all patients who had their catheter inserted (since 2006) within the Geelong regional service. We compared the outcomes of percutaneous catheter insertion under image guidance (percutaneous group, *n* = 29) with the laparoscopic catheter placement technique (laparoscopic group, *n* = 61). Perioperative, follow‐up and catheter outcome data were collected. There were no exclusion criteria. Analysis was performed using the unpaired student *t*‐test and chi‐squared test.

**Results:**

Ninety catheters were inserted between 2006 and 2017 in mostly male patients (63%) with a mean age of 60 ± 0.4 years. The most common aetiology of chronic kidney disease was diabetic nephropathy (34%). Percutaneous insertion required less operative time, shorter hospital stay and earlier initiation of peritoneal dialysis. In the longer term, percutaneous catheters were more likely to migrate and laparoscopic catheters were more durable but more often associated with peritonitis. Thirty‐day complication rates did not differ between both groups. No Clavien‐Dindo grade 3 or 4 complication was reported.

**Conclusions:**

Laparoscopic insertion of peritoneal dialysis catheters at our centre is performed safely and with patient outcomes comparable to published literature. Percutaneous insertion represents a safe and effective alternative based on the study findings.

## Graphical abstract

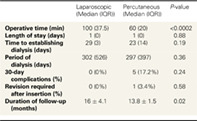

Comparison of Laparoscopic v. Percutaneous insertion of Peritoneal Dialysis Catheters

## Introduction

Chronic kidney disease represents a significant health burden affecting up to 13% of Australians.^1^ Some will progress to end stage disease requiring renal replacement therapy including peritoneal dialysis. Effective peritoneal dialysis requires the placement of an indwelling peritoneal dialysis (PD) catheter into the peritoneal cavity. The insertion can be done by a surgical (open or laparoscopic) or a percutaneous (with or without fluoroscopy/ultrasound) approach. Surgical insertion is usually performed by general or transplant surgeons but at our regional centre (Geelong, Australia) these catheters are inserted by a single urological surgeon. We report the outcomes of laparoscopic and percutaneous insertion of PD catheters in our centre and compare our findings with the published literature.

## Method

### Literature search

A literature search was conducted using PUBMED and Cochrane Library. The search was filtered to include all studies from the year 2000 to June 2018 using a combination of MeSH terms and phrases including: percutaneous Tenckhoff catheter insertion, laparoscopic Tenckhoff catheter insertion, Tenckhoff catheters, surgical insertion of Tenckhoff catheters and peritoneal dialysis. We also manually searched the reference lists of all the studies identified.

### Procedure protocol and outcome measures

The medical records of all patients who had their PD catheters inserted percutaneously and/or laparoscopically by the Geelong regional service between April 2006 and November 2017 were reviewed retrospectively. There were no exclusion criteria. Data including patient demographics, perioperative (including post‐operative complications) and follow‐up data were collated using an Excel spreadsheet. Patient and catheter‐related outcomes were recorded at 30 days post insertion, 365 days post insertion and as at November 2017. Time to dialysis was defined as the time (days) following catheter insertion to patient commencing PD for renal replacement therapy. Post‐operative complications were defined as adverse events occurring within 30 days of catheter insertion and graded by the Clavien‐Dindo classification.^2^ Follow‐up time was calculated from the day of catheter insertion to either November 2017, patient's death (during follow‐up period), or date of catheter removal, whichever was earlier. Catheter obstruction was the inability to exchange dialysis fluid. Failed peritoneal dialysis was defined as a decline in renal function despite dialysis.

### Patient selection for type of catheter insertion

Patients were referred for either laparoscopic or percutaneous insertion of catheters based on nephrologist preference. Factors that favoured percutaneous insertion included the longer wait time for laparoscopic catheters and patients that were not fit for a general anaesthetic. Over time, percutaneous catheters became the preferred option due to ease of access and the shorter waiting times. All patients referred for percutaneous insertion was accepted.

### Method of catheter insertion

#### Laparoscopic method

Under general anaesthesia patients were positioned in Trendelenburg under general anaesthesia with an indwelling catheter *in situ*. Prophylactic intravenous antibiotics were administered prior to procedure. A 2 cm paramedian incision centred 3 cm lateral to and 3 cm caudal to the umbilicus was made for the camera port. Two 5 mm working camera ports were placed under vision on the opposite side of the abdomen 12–15 cm apart to achieve triangulation. Adhesions if present were divided. The small and large bowel were mobilized and retracted cephalad to provide access to the most dependent part of the peritoneal cavity. The PD catheter (Argyle 15Fr PD catheter, Covidien, MA, USA) was passed into the recto‐vesical or recto‐vaginal pouch and secured with a purse‐string suture to the posterior bladder wall with the tip sitting as dependent as possible. The pneumoperitoneum was released, and camera ports removed. The catheter was drawn out of the peritoneal cavity using a 2/0 polyglactin 910 suture tied to the distal end of the catheter. The inner cuff was secured to the peritoneum using a 4/0 polydioxanone suture and anterior rectus fascia is closed. The catheter was tunnelled subcutaneously to a pre‐marked exit site with the outer cuff sited in the subcutaneous fat. The wounds were closed with subcuticular sutures and dressed with a transparent occlusive dressing. The catheter was flushed and aspirated to check inflow and outflow patency.

#### Percutaneous modified Seldinger technique under fluoroscopic guidance

Under local anaesthesia and sedation in the supine position the PD catheter was inserted under fluoroscopic and ultrasound guidance by a radiologist in the fluoroscopy suite. Under ultrasound guidance, access to the abdominal iliac fossa cavity was gained through a micropuncture and a guide wire placed. Through a combination of a 5Fr dilating sheath and 24Fr peel away sheath the 15Fr Argyle double cuff PD catheter was inserted. The inner cuff was positioned at the edge of peritoneum using ultrasound guidance. The catheter was flushed and aspirated to check inflow and outflow patency.

### Protocol for commencing dialysis

The timing of commencing dialysis following PD catheter insertion was at the discretion of the dialysis nurse specialist. Dialysis was commonly delayed in laparoscopic catheters due to a perception that the larger incision is more likely to leak but this perception changed over time as understanding improved. Patients with rapidly worsening renal function were started earlier on dialysis.

### Statistical analysis

Data were recorded as percentages or mean ± *SEM* unless stated otherwise. Continuous variables were tested for normality by assessment of the histogram and analysed using the unpaired student *t*‐test. Non‐parametric data were compared using the Mann–Whitney U‐test and the chi‐squared test. Statistical significance defined where *P* < 0.05.

## Results

The first laparoscopic PD catheter inserted at our centre was in 2006, and the percutaneous technique was introduced in 2014 with a total of 90 inserted as of November 2017. Of the 90 catheters inserted, 61 were inserted laparoscopically by a single urological surgeon, and 29 were inserted percutaneously by a single interventional radiologist. Most were for primary (first) insertion of PD catheter but existing catheters were revised (re‐inserted or re‐positioned) laparoscopically in 8 patients and percutaneously 1 patient (*P* = 0.15). Follow‐up was longer in the laparoscopic cohort given that laparoscopic catheters had been inserted over a longer period (29.7 ± 3.3 vs. 13.8 ± 1.5 months). A majority of patients were male while those in the percutaneous cohort had a higher body mass index (Table 1).

**Table 1:** Patient demographics

	Laparoscopic (*n* = 61) (Mean ± *SD*)	Percutaneous (*n* = 29) (Mean ± *SD*)	*P*‐value
Age (years)	59.3 ± 15.9	59.0 ± 15.0	0.87
Male	40 (65.6%)	17 (58.6%)	0.52
Body mass index (kg/m^2^)	25.3 ± 4.6	28.0 ± 4.2	0.01
Previous abdominal surgery	24 (39.3%)	10 (34.5%)	0.66
Previous PD catheter insertion	8 (13.1%)	1 (3.4%)	0.15

The percutaneous technique was quicker than the laparoscopic technique and patients left the hospital earlier despite having a higher body mass index (Table 2). Patients could also be established on their full dialysis protocol in half the time following percutaneous insertion. However, catheters inserted laparoscopically remained in use for over twice as long as those inserted percutaneously (818.6 ± 94.6 days compared with 314 ± 47.7 days, *P* < 0.0001). This was not seen in the subgroup analysis comparing catheters inserted over the same period of time (see following section).

**Table 2:** Laparoscopic (*n* = 61) and percutaneous (*n* = 29) insertion of PD catheter since 2006

	Laparoscopic (Median (IQR))	Percutaneous (Median (IQR))	*P*‐value
**Operative time (min)**	90 (60)	60 (20)	<0.0001
**Length of stay (days)**	1 (1–2)	1 (1–1)	0.026
**Time to establishing dialysis (days)**	32 (27.25)	23 (14)	0.009
**Period of dialysis (days)**	656.5 (1110.5)	297 (397)	0.003
**Thirty‐day complications (%)**	10 (16.4%)	5 (17.2%)	0.86
**Revision required after insertion (%)**	7 (11.5%)	1 (3.4%)	0.21
**Duration of follow‐up (months)**	29.7 ± 3.3	13.8 ± 1.5	<0.0001

### Post‐operative complications

Post‐operative 30‐day complication rates were comparable between cohorts but more patients developed abdominal ileus following laparoscopic insertion (Table 2). Exit site leakage (bleeding or oozing) was more common in those who had their catheters inserted percutaneously. As detailed in Table 3, complications in the laparoscopic cohort were Clavien‐Dindo Grade 1 (nausea/vomiting, ileus and pericatheter leakage) and only two patients had a Grade 2 complication (exit site infection requiring antibiotics).^2^ Two further patients had a Grade 3b complication as they required revision of their PD catheter under general anaesthesia.

**Table 3:** Comparison of procedure outcomes and complications following insertion of PD catheters between our study and published studies

	Our study		Voss, *et al*. †		Abdel Aal, *et al*.		Al–Hwiesh, *et al*. ††	
Insertion technique	Laparoscopic	Percutaneous	Laparoscopic	Percutaneous	Laparoscopic	Percutaneous	Laparoscopic	Percutaneous
**N**	61	29	56	57	190	50	25	27
**Follow up (months)**	84.7 ± 2.4	19.1 ± 3.5*	‐	‐	‐	‐	12 ± 5	13 ± 3
**Age (years)**	59.3 ± 3.3	58.9 ± 2.7	60.8	61.1	54	56	52 ± 12.3	48 ± 15.2
**Operative time (minutes)**	90 (60–120) ‡	60 (40–60) ‡	48 (40–55) ‡	55 (45–60) ‡, *	‐	‐	‐	‐
**Length of stay (days)**	1 (1–2) ‡	1 (1–1) ‡, *	1.04 (0.83–2.0) §	1.04 (0.88–1.88) §	‐	‐	‐	‐
**Time to dialysis (days)**	43.1 ± 4.6	25.6 ± 3.0*	‐	‐	‐	‐	15 ± 6.2	6 ± 3.4*
**Complications:**								
Overall	10/61 (16.4%)	5/29 (17.2%)	‐	‐	119/190 (63%)	31/62 (50%)	12/21 (57%)	11/22 (50%)
**Migration**	1/61 (1.6%)	1/29 (3.4%)	‐	‐	‐	‐	‐	‐
**Nausea/vomiting**	2/61 (3.3%)	0						
**Exit site infection**	2/61 (3.3%)	0	17/56 (30.4%)	14/57 (24.6%)	10/190 (5.2%)	2/50 (4%)	2/25 (8%)	2/27 (7.4%)
**Peritonitis**	0	0	24/56 (42.9%)	16/57 (10.7%)	32/190(16.8%)	8/50 (16%)	1/25 (4%)	1/27 (3.7%)
**Pericatheter leakage**	1/61 (1.6%)	1/29 (3.4%)*	3/56 (5.4%)	1/57 (1.8%)	6/190 (3.1%)	5/50 (10%)*	4/25 (16%)	1/27 (3.7%)*
**Catheter failure**	1/61 (1.6%)	1/29 (3.4%)	‐	‐	‐	‐	3/25 (12%)	2/27 (7.4%)
**Hernia formation**	0	0	8/56 (14.3%)	4/57 (7%)	9/190 (4.7%)	3/50 (6%)	0	0
**Ileus**	3/61 (4.9%)*	0	‐	‐	‐	‐	‐	‐
**Bleeding/oozing around site**	0	2/29 (6.9%)*	‐	‐	‐	‐	0	0
**Death**	0	0	6/56 (10.7%)¶	4/57 (7%)¶	‐	‐	0	0

Data are shown as mean ± *SEM* or number (%) unless otherwise specified. ‐ data not included in study.

* *P* <0.05.

^†^ Study reported complication rates at 365 days follow up.

^‡^ Median (Interquartile range).

^§^ Days to hospital discharge (interquartile range).

^¶^ Death from any cause during follow‐up.

^††^ Early complications included only (study defined it as within 2 weeks of insertion).

In the percutaneous cohort, complications were Grade 1 (pericatheter leakage and bleeding/oozing that settled with conservative management) but two out of five patients (40%) of complications were grade 3b requiring revision of the PD catheter under general anaesthesia. These findings are compared with other published studies in Table 3.

### Long term outcomes

**Fig. 1:**
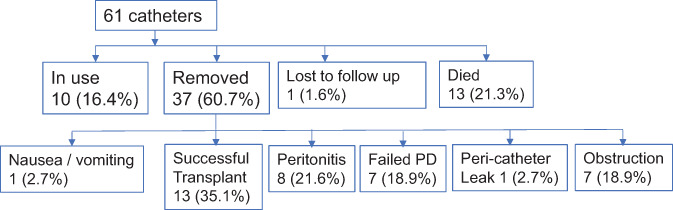
Long‐term outcomes of laparoscopically inserted PD catheters (*n* = 61) from 2006 till November 2017.

**Fig. 2:**
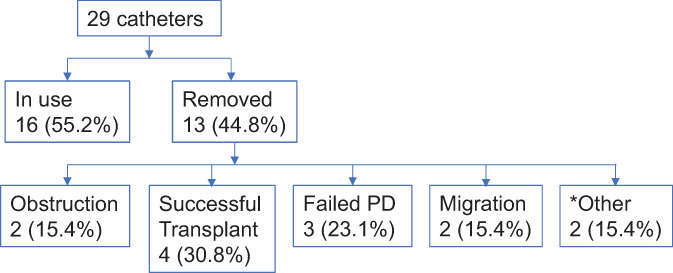
Long term outcomes of percutaneously inserted PD catheters (*n* = 29) from 2014 till November 2017. *One catheter was removed as the patient developed peritonitis from perforated rectal cancer. Another catheter was removed as the patient's renal function recovered.

Duration of follow‐up was 29.7 ± 3.3 months in the laparoscopic cohort compared with 13.8 ± 1.5 months in the percutaneous cohort (Table 2, *P* < 0.0001). Outcomes for both cohorts are described by Figures 1 and 2. Over the long term, PD catheters inserted percutaneously were more likely to migrate leading to obstruction and failure requiring removal (2 *vs*. 0 patients, *P* = 0.04). Eight patients with laparoscopically inserted PD catheters developed peritonitis (*P* = 0.04). At the end of follow‐up 16 out of 29 patients in the percutaneous cohort (55.2%) continued to use their PD catheter compared to 10 out of 61 patients in the laparoscopic cohort (16.4%).

### Sub‐group analysis

Outcomes for both cohorts over the same period (2014–2017) were compared (Table 4). The number of percutaneous PD catheters inserted surpassed laparoscopic catheters from 2016 (83% of insertions). Percutaneous catheters required less operative time but took a similar time to establish dialysis. Length of stay, duration on dialysis and the frequency of post‐operative complications were similar. One patient required revision following percutaneous PD catheter insertion between 2014 and 2017, whereas no patients in the laparoscopic group required revision of their catheter.

**Table 4:** Comparison of laparoscopic (*n* = 9) and percutaneous (*n* = 29) insertion of PD catheters from 2014 to 2017

	Laparoscopic (Median (IQR))	Percutaneous (Median (IQR))	*P*‐value
**Operative time (min)**	100 (37.5)	60 (20)	<0.0002
**Length of stay (days)**	1 (0)	1 (0)	0.88
**Time to establishing dialysis (days)**	29 (3)	23 (14)	0.19
**Period of dialysis (days)**	302 (526)	297 (397)	0.36
**30‐day complications (%)**	0 (0%)	5 (17.2%)	0.24
**Revision required after insertion (%)**	0 (0%)	1 (3.4%)	0.58
**Duration of follow‐up (months)**	16 ± 4.1	13.8 ± 1.5	0.02

**Fig. 3:**
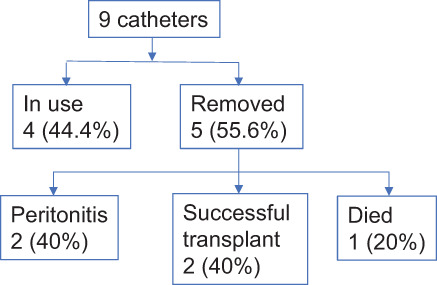
Long term outcomes of laparoscopically inserted PD catheters (*n* = 9) from 2014 till November 2017.

The number of catheters still in use at the end of follow‐up was similar for both cohorts (Figs. 2 and 3). PD catheters were primarily removed following renal transplantation although laparoscopic PD catheters were more likely to be removed following peritonitis compared with percutaneous catheters.

## Discussion

The present study shows that PD catheters can be safely inserted by a urological surgeon practising in a regional centre. Outcomes of the procedure and complication rates were broadly comparable to reported studies. Similarly, complications were similar when we compared laparoscopic PD catheter insertion to percutaneous insertion with advantages specific to each modality. In our cohort the percutaneous technique was quicker and patients were discharged from hospital earlier. Patients also started dialysis earlier following percutaneous insertion. However, most of these advantages were not seen in the sub‐group analysis comparing more recent catheter insertion over the same period (2014–2017). We postulate that the slightly poorer early results may suggest a steeper learning curve for laparoscopic insertion compared to percutaneous insertion.

To date, a plethora of studies have compared surgical insertion of PD catheters to percutaneous insertion. Unsurprisingly, study design and surgical technique were not been standardized.^3^ Patients selected for percutaneous catheter insertion were also commonly subject to strict inclusion criteria. Surgical technique varied significantly between open or laparoscopic surgical insertion^4^, ^5^ while many percutaneous insertions of PD catheters did not utilize fluoroscopic or ultrasound guidance.^6^, ^7^ Image guidance is the recommended gold standard for percutaneous insertion of PD catheters.

Our study included all patients referred for insertion of PD catheter and did not exclude obese patients, those with previous abdominal surgery or those requiring revision of catheters. Contemporary laparoscopic techniques were employed including selective adhesiolysis.^8^ Catheter tips were sutured in the pelvis during laparoscopy to prevent migration.^9^ Similarly, fluoroscopy was routinely used for percutaneous catheter insertion to minimize morbidity and to confirm catheter position. Our results demonstrate the success of this approach with no severe (Clavien‐Dindo Grade 3 or higher) 30‐day complications. Post‐operative ileus was more common following laparoscopic insertion while exit site bleeding that settled without re‐operation was more common in the percutaneous cohort.

We compared perioperative end points (procedure time and hospital stay) and found that the percutaneous technique was faster with a shorter hospital stay although these findings changed in the subgroup analysis (see below). The percutaneous technique took longer in the New Zealand study^10^ (Table 3). Time to dialysis has previously been shown to be faster after percutaneous insertion.^6^ Both were prospective studies but obese patients and those with previous abdominal surgery were excluded.

Only one other study compared laparoscopic with percutaneous insertion in a similar cohort.^11^ Two hundred and forty patients including obese patients and those with a history of abdominal surgery were recruited. Patients who had a catheter placed after more than one attempt (not clearly defined) were excluded. No difference in complication rates or catheter survival between techniques was shown. Perioperative end points were not reported.

Outcomes of both techniques have not previously been compared over the long term. We followed our patients for an extended period (29.7 ± 3.3 months in the laparoscopic cohort, 13.8 ± 1.5 months in the percutaneous cohort). Over this time, percutaneous PD catheters were more likely to migrate (*P* = 0.04) possibly because the catheters were not fixed in the pelvis. The higher incidence of catheter migration did not translate to the higher risk of abdominal viscus perforation reported previously.^7^, ^12^ Peritonitis was more common following laparoscopic insertion^3^, ^4^, ^10^, ^12^, ^13^ and may relate to the longer operative time and the size and number of incisions required. Most episodes of peritonitis in the present study occurred greater than 30 days post‐procedure and may relate to other factors such as poor patient technique while performing PD.

PD catheters at our centre were inserted by laparoscopy for 8 years before the percutaneous technique was introduced. The significant difference in length of follow‐up (29.7 ± 3.3 *vs*. 13.8 ± 1.5 months, *P* < 0.0001) may have biased our findings. Therefore, both cohorts between 2014 and November 2017 were compared in a sub‐group analysis. The laparoscopic cohort still had a slightly longer follow‐up but all outcomes from the primary analysis were now equal except for a shorter procedure time for percutaneous insertion. Within 2 years however, the percutaneous technique accounted for 80% of all PD catheter insertions and became the preferred referral option for nephrologists. A likely explanation is the long surgical waiting list and difficulty in securing access to the operating suite.

Our study reports one of the largest cohorts comparing laparoscopic to percutaneous PD catheter insertion in the published literature. Although retrospective, data were extracted from a customized electronic medical record system used by the Renal Dialysis service. This ensured a high level of accuracy and record keeping due to the close follow‐up required for patients on renal dialysis. Few patients were lost to follow‐up as we are the only dialysis unit for our regional health service. Each procedure was performed by a single surgeon or radiologist thereby reducing inter‐operator variability. These strengths minimize the selection and information bias in the study.

Despite the above strengths, the retrospective nature of the study suggests that selection bias and information bias could not be fully compensated for. However, no patients were excluded from the study in order to minimize selection bias. Despite that, the body mass index of patients undergoing percutaneous insertion was slightly higher. The difference is unlikely to be clinically significant because patients in both cohorts were overweight. The length of follow‐up was different for both cohorts and therefore a sub‐group analysis (comparing both cohorts over the same period) was performed.

## Summary

In conclusion, laparoscopic PD catheters can be safely inserted by a urological surgeon practising in a regional centre. However, percutaneous PD catheters can be inserted more quickly but over the long term, the catheters are more likely to migrate and fail. The risks of post‐operative ileus and peritonitis are higher following laparoscopic insertion. Further data from large, well designed, prospectiverandomized controlled trials are necessary to confirm these findings. Comparison of the cost‐effectiveness of each technique would also be greatly informative.

## Conflict of interest

None declared.
